# Human resources for health care delivery in Tanzania: a multifaceted problem

**DOI:** 10.1186/1478-4491-10-3

**Published:** 2012-02-22

**Authors:** Fatuma Manzi, Joanna Armstrong Schellenberg, Guy Hutton, Kaspar Wyss, Conrad Mbuya, Kizito Shirima, Hassan Mshinda, Marcel Tanner, David Schellenberg

**Affiliations:** 1 Ifakara Health Institute, Health System and policy thematic, Kiko Ave 463, Mikocheni, P.o. Box 78373, Dar es Salaam, Tanzania; 2 Ministry of Health, P.o. Box 9083, Dar es salaam, Tanzania; 3 Department of Disease Control, London School of Hygiene and Tropical Medicine, Keppel Street, Bloomsbury, London WC1E 7HT, London, UK; 4 Swiss Tropical & Public Health Institute Socinstrasse 57, P.o. Box CH - 4002, Basel, Switzerland; 5 University of Basel, Petersplatz 1, CH-4003, Basel, Switzerland

## Abstract

**Background:**

Recent years have seen an unprecedented increase in funds for procurement of health commodities in developing countries. A major challenge now is the efficient delivery of commodities and services to improve population health. With this in mind, we documented staffing levels and productivity in peripheral health facilities in southern Tanzania.

**Method:**

A health facility survey was conducted to collect data on staff employed, their main tasks, availability on the day of the survey, reasons for absenteeism, and experience of supervisory visits from District Health Teams. In-depth interview with health workers was done to explore their perception of work load. A time and motion study of nurses in the Reproductive and Child Health (RCH) clinics documented their time use by task.

**Results:**

We found that only 14% (122/854) of the recommended number of nurses and 20% (90/441) of the clinical staff had been employed at the facilities. Furthermore, 44% of clinical staff was not available on the day of the survey. Various reasons were given for this. Amongst the clinical staff, 38% were absent because of attendance to seminar sessions, 8% because of long-training, 25% were on official travel and 20% were on leave. RCH clinic nurses were present for 7 hours a day, but only worked productively for 57% of time present at facility. Almost two-third of facilities had received less than 3 visits from district health teams during the 6 months preceding the survey.

**Conclusion:**

This study documented inadequate staffing of health facilities, a high degree of absenteeism, low productivity of the staff who were present and inadequate supervision in peripheral Tanzanian health facilities. The implications of these findings are discussed in the context of decentralized health care in Tanzania.

## Background

In the last decade developing countries have witnessed an unprecedented increase in funds for the procurement of commodities such as drugs, vaccines and other medical supplies through the Global Fund for HIV/AIDS, Tuberculosis and Malaria (GFATM), Global Alliance for Vaccine Initiatives (GAVI) and other Global Health Initiatives (GHIs). At the same time there is growing recognition of local health system constraints which impair the efficient delivery of health care and threaten to reduce the effectiveness of the GHIs [1-5]. Scale-up of basic health services depends on the availability of key health systems inputs such as human resources, infrastructure, equipment, drugs, finance, information and governance. Where the available infrastructure and human resources are used in an efficient way and are fully utilized, then the introduction or scale-up of additional interventions will require additional health workers, drugs, equipment and buildings. However, if there is inefficient use of available resources, productivity gains may be possible through enhanced efficiency.

The ratio of health workers to population has a direct relationship with survival of women during childbirth and children in early infancy: as the number of health workers declines, survival declines proportionately [6]. Most sub-Saharan countries face human resource shortages for health service delivery [3,7]. While the world average for health worker (clinical staff, nurses and all types of health workers) density per 1000 population is 9.3, there is marked inequality with 18.9 health workers per 1000 population in Europe and only 2.3 in Africa [7]. There is also marked variation within Africa: in Chad there are 0.16 nurses per 1,000 population, and Tanzania has 0.39 nurses and 0.25 clinical staff (medical doctors, assistant medical officers and clinical officers) per 1000 population [8]. In Tanzania, on average there is one prescriber (generally mid level providers trained in-country, rather than medical doctors) in each primary facility with the workload averaging 29 outpatients per clinician per day in health centres and 20 in dispensaries [9]. Marked inequalities in the distribution of health workers are documented in Tanzania in terms of per capital distribution and rural urban imbalances [10,11]. While the average is 1.4 health workers per 1000 people in the country, this varies greatly between districts, from 0.3 per 1000 in Bukombe district to 12.3 per 1000 in Moshi district [11]. The health worker shortage in Africa has been attributed to low output of new health workers by medical schools, out migration to other sectors and to more lucrative countries because of retention related factors including poor remuneration and adverse working conditions at home [12,13]. HIV/AIDS has both increased demand for skilled health workers and directly reduced their availability [14]. There is also an urban-rural imbalance of health workers with more staff in urban centres [12].

The efficient functioning of any health system is contingent on the productivity of the workforce. How best to measure productivity is context specific but generally requires bench marks based on duties defined in a job description. Performance indicators are then compared against targets [15]. However job descriptions are not widely available and performance indicators not generally agreed in Tanzania. A fair and accurate employee performance review may begin with tracking employee behaviors and patterns [16]. There is evidence that productivity of health staff in developing countries is sub-optimal and that personnel are under-utilized [17,18]. For example, in one study from Cameroon only 27% of health workers' time was spent on productive activities (curative and clinical work) [18] and in Tanzania the estimated time health workers spent on productive activities was 57% [8]. Potential productivity gains of existing staff were estimated at approximately 26% in Tanzania and 35% in Chad [8]. Various solutions to increase staff and productivity have been proposed that include improved management measures, specific training tailored to the local area, strengthening of enabling factors such as equipment and skills, and the introduction of financial incentives to increase workers' efforts [2,19-21].

We conducted a number of health system assessments in southern Tanzania as part of an evaluation of Intermittent Preventive Treatment in infants (IPTi) [22,23]. A structural and functional assessment of the health system [5] preceded IPTi implementation by routine health services [24] and monitoring of costs [25]. Here we report the analysis of the multifaceted human resource problems in terms of staffing levels in comparison with the Ministry of Health's guidelines, the extent of absenteeism and productivity challenges in peripheral health facilities. We define absenteeism as a habitual pattern of absence from a duty or obligation, including both a fair pattern such as health workers' leave, and a separate and managerially-addressable pattern such as health workers attending training, collecting salaries, supplies or drugs.

## Methods

### Study area

The study was conducted in the five districts of Nachingwea, Lindi Rural, Ruangwa, Tandahimba and Newala Districts in Southern Tanzania, with a total population of about 900,000 in 2002. A detailed description of the area is given elsewhere [5]. Briefly, the public health system comprises a pyramidal network of dispensaries, health centres and hospitals. Some villages have volunteer village health workers. The national policy requires that children under the age of five and pregnant women are exempted from fees at government health facilities. However, in practice they pay for drugs and supplies when they are out of stock at the facility. The area is characterized by the highest child mortality in Tanzania; under-five mortality was 153/1000 in the ten year period preceding a 2004/5 Demographic and Health Survey [26].

The health system in Tanzania is largely decentralized [27]. The district is empowered to set priorities, and is responsible for health service implementation and for supervision of individual health facilities on a monthly basis. The dispensary is the most peripheral level of service delivery, catering for between 6,000 to 10,000 people. Health centres are expected to serve about 50,000 people, approximately the population of one administrative division, providing in-patient services for patients referred from lower levels. Higher up the service pyramid, each district is supposed to have a district hospital. Where there is no government hospital, an available faith-based or NGO hospital is often designated as the district hospital. The regional hospital offers services similar to those at district level but has specialists in various fields and offers additional services not available at district hospitals. The national referral hospital is the highest level of inpatient services.

The Ministry of Health established recommendations for staffing levels in the different types of health facility. Two clinicians and two nurses are recommended for each dispensary and four clinicians and nine nurses for each rural health centre [28]. Health workers delivering the majority of care in rural primary health facilities (dispensaries and health centres) are generally "clinical staff" (Assistant Medical Officers or Clinical Officers or Assistant Clinical Officers) or nurses; there are no medical doctors. Clinical staff attends four or six years of secondary education before three years of professional training. Nurses include Nursing Officers, Nurse Midwives, Public Health Nurse 'A' and 'B' and Maternal and Child Health Aides (though this latter cadre is being phased out); their training involves four years of secondary education followed by three years of professional training. However, because of health worker shortages, it is not uncommon to find auxiliary nursing staff with only basic primary education of 7 years and a single year's introduction to nursing courses performing the tasks of a trained nurse.

### Study design and data Collection

Multiple methods were employed including a health facility survey, in-depth interviews and a time and motion study. Purposive sampling was used. Data quality assurance for each method is explained under respective sub-section that follows. Ethical approval was received from local and national institutional review boards (Ifakara Health Institute and the National Tanzania Medical Research Co-coordinating Committee) through COSTECH (Tanzania Commission for Science and Technology). During data collection in health facilities, verbal consent was sought from participating health workers.

### Health facility survey

A baseline health facility survey was conducted in September 2004 to facilitate the planning for implementation of IPTi and familiarization with the local health system. All 134 health facilities in the five districts were visited including hospitals, health centres and dispensaries of the public health care system, non-governmental not-for-profit organizations and the private sector. Using a modular tool, data were collected on (i) the number and cadre of health workers employed at the facility and (ii) the number actually present on the specific day of the survey. Other modules assessed the availability of equipment and supplies. Staff was asked about their main activities, reasons for their colleagues' absence, and supervision by district health staff, the functioning of vaccination activities and their views on how to improve services.

Training of experienced field workers was carried out over a period of five days and included interview technique, group work, role-play and practical fieldwork as well as a pilot test of the survey instruments. The survey was conducted by 16 interviewers working in one to two facilities each day in groups of two, forming eight teams, with two supervisors who assisted the survey co-ordinator. A letter of introduction from each Council Health Management Team, signed by the District Medical Officer and the District Executive Director, was given out at each facility and verbal consent sought before proceeding with interviews.

To help assure the quality of data, at least one interview was accompanied by a supervisor each day. All forms completed each day were reviewed in the evening and feedback given to the interview teams before the next day's work. Data was collected using conventional paper forms which were double entered into DMSys software (Microsoft^® ^Visual FoxPro^® ^platform, Cincinnati, USA), followed by checks of range and resolution of any inconsistencies. Analysis was done using Stata ^© ^(version 8, College Station, Texas, USA).

### In-depth Interviews

These were done with nurses at RCH clinics to explore their perception of work load. In comparison areas, where IPTi had not been introduced, the discussion focussed on how difficult would it be to implement a new preventive malaria intervention "IPTi" linked to vaccination. In intervention area nurses were asked how difficult was it to implement IPTi? To ensure data quality, data were collected by experienced field interviewers who were trained for two weeks. The training included lectures, group discussions, field practices and feedback sessions. The survey coordinator visited each team to observe activities and discuss practical concerns.

### Time and motion study

A time and motion study was done in 24 dispensaries and health centres in the project area during November-December 2005 [24]. Briefly, the objective was to document health workers time use at a Reproductive and Child Health (RCH) clinic. Pairs of interviewers spent a week at each participating health facility and time and motion data were collected towards the end of their stay, when the staff had grown used to the presence of the interviewers. Nurses delivering EPI vaccines and other interventions integrated at the RCH clinic were followed on a typical vaccine clinic day as vaccination is not done every day. Two major categories of time use were distinguished, namely productive and non-productive time. Productive time activities included room cleaning, other work room preparations, mothers' education sessions, delivery of interventions like family planning, provision of *sulphadoxine-pyrimethamine *(SP) to pregnant women (IPTp) and infants (IPTi), recording doses and dates in immunization cards and in Health Management Information System (HMIS). Non-productive activities included unexplained breaks, social contacts and waiting for patients. This non-productive time - through improved staff management and accountability - can be potentially translated into productivity gains leading to improved health service provision. Data were entered at the point of collection using a personal digital assistant (PDA) [29]. The PDA had a menu of nurses' activities; when an activity was selected the time was automatically recorded as the start time for that activity and the end time for the previous activity. The device allowed immediate checking of ranges and data consistency. Analysis was done using Stata ^© ^(version 8, College Station, Texas, USA).

## Results

### Health workers at peripheral health facilities

A total of 134 health facilities were surveyed in the five districts; one facility was closed. Of those surveyed, 127 were primary facilities (health centres and dispensaries) and seven were hospitals. During analysis one regional hospital was dropped as it serves several districts. As shown in Table 1 the study documented clear lines of responsibility for clinical staff and nurses in primary facilities. The average is age of staff is 44 years and 15 years of working at the facility. The vast majority (94%) of clinical staff reported their primary task as case management of patients, though a minority (5%) said their main activity was administration. Nurses reported a broader range of primary activities, dominated by vaccination (33%), antenatal care (23%), case management (16%), and nursing procedures (14%).

**Table 1:** Health workers primary task in primary health facilities in southern Tanzania

Primary Task	Clinical staff N = 82	Nurses N = 81
	%	%
Case management of patients	94 (n = 77)	16 (n = 13)
Administration	5 (n = 4)	3 (n = 2)
Vaccination and all child preventive services	1 (n = 1)	33 (n = 27)
Nursing procedures	0	14 (n = 11)
Childbirth care	0	11 (n = 9)
Antenatal	0	23 (n = 19)

The Ministry of Health and Social Welfare's (MOHSW) staff guideline recommends 441 clinical staff and 854 nurses for the facilities visited [28]. However, only 20% (90/441) of the recommended number of clinical staff and 14% (122/854) of the recommended number of nurses had been employed (Table 2). This equates to an overall staffing level of 0.10 clinical staff per 1000 population and 0.14 nurses per 1000 population. There was marked variation in staffing levels between districts, ranging from 0.05 - 0.16 per 1000 population for clinical staff and 0.07-0.23 per 1000 population for nurses.

**Table 2:** Health workers Density per District in health facilities in southern Tanzania compared to Ministry of Health guideline

Descriptions		Clinical Staff								Nurses							
		
		Rec^a^		Employed			Available on the day of survey			Rec^a^		Employed			Available on the day of survey		
**District**	**Popn^b^**	***No***	***Per 1000 Popn^b ^Equiv***	***No***	***% Rec***	***Per 1000 Popn^b ^Equiv***	***No***	***% Emp^c^***	***Per 1000 Popn^b ^Equiv***	***No***	***Per 1000 Popn^b ^Equiv***	***No***	***% Rec***	***Per 1000 Popn^b ^Equiv***	***No***	***% Emp***	***Per 1000 Popn^b ^Equiv***

Lindi Rural	214,882	115	0.54	24	21%	0.11	19	79%	0.09	194	0.90	32	16%	0.15	16	50%	0.07

Nachingwea	161,473	119	0.74	22	18%	0.14	11	50%	0.07	271	1.68	24	09%	0.15	11	46%	0.07

Ruangwa	124,009	57	0.46	20	35%	0.16	3	15%	0.02	116	0.94	29	25%	0.23	15	52%	0.12

Newala	183,344	77	0.42	13	17%	0.07	8	62%	0.04	136	0.74	22	16%	0.12	12	55%	0.07

Tandahimba	203,837	73	0.36	11	15%	0.05	9	82%	0.04	137	0.67	15	11%	0.07	8	53%	0.04

All districts	887,545	441	0.50	90	20%	0.10	50	56%	0.06	854	0.96	122	14%	0.14	62	51%	0.07

^a^Rec: Recommended health worker as per Ministry of Health Guideline 1999

^b^Popn: population

^c^Emp: employed

There was a high level of absenteeism amongst employed staff, with 44% of clinical staff and 49% of nurses absent from their work station on the day of the survey. This reduced the effective coverage of staff to 0.06 and 0.07/1000 population for clinical staff and nurses respectively. Table 3 shows that 38% of the absent clinical staff and 29% of absent nurses were attending meetings or short-term training seminars, 25% of both cadres were on official travel (collecting vaccines, drugs or wages from the district offices) and 20% were on leave.

**Table 3:** Reasons for health workers absence in primary facilities

Reason for absence	Clinical staff N = 40	Nurses N = 45
	**%**	**%**
Meetings/Seminars	38 (n = 15)	29 (n = 13)
Other official travel*	24 (n = 10)	25 (n = 11)
On leave	20 (n = 8)	20 (n = 9)
Long term training	8 (n = 3)	4 (n = 2)
On a different shift	5 (n = 2)	4 (n = 2)
Outreach	0 (n = 0)	2 (n = 1)
Sick	3 (n = 1)	9 (n = 4)
Other	2 (n = 1)	7 (n = 3)

* Collecting vaccines, drugs or wages from the district offices

### Activities and Time Use

**Figure 1:**
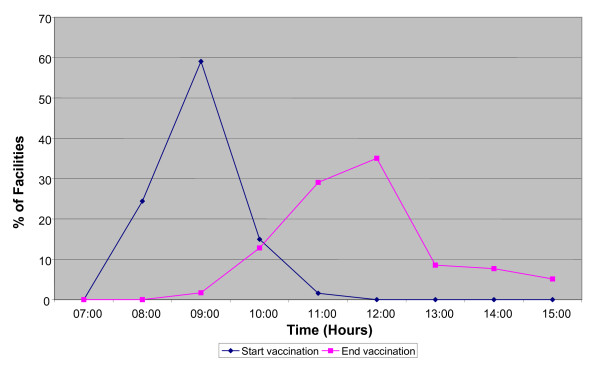
**Timing of vaccination activities in primary health facilities**.

Vaccination activities in primary facilities were concentrated in the morning hours of the working day (Figure 1). The peak starting time was around 9:00am and completion time was around 12:00 noon. Congestion at clinics was common during these times as we observed nurses encourage people to come early for most health services leading to a concentration of activities in the morning hours. Only a few activities, such as family planning, continue into the afternoon. Table 4 shows the results of the time and motion study. Out of the 24 facilities visited, 19 had vaccination activities during the researchers' visits. RCH nurses spent an average of 7 hours 9 minutes per day at their health facility, of which 4 hours 3 minutes were considered productive. An average of 1 hour 30 minutes was spent administering EPI vaccines or other child health interventions linked to vaccination (such as vitamin A, IPTi), and filling the health Management Information System (HMIS) forms. Specifically, HMIS took an average of 26 minutes (range 3-101 minutes). A further 59 minutes were spent on antenatal care and family planning. Nurses in eight facilities were occupied with case management for a mean of 29 minutes. Other activities (non contact productive activities including work place cleaning and preparation of work day supplies) took 1 hour and 10 minutes of nurse's time. Over half (56% (10/18)) of the nurses were unproductive for three or more hours, waiting for patients, chatting or just wandering around. Unexplained absenteeism accounted for 51 minutes on average per nurse.

**Table 4:** Time spent on specific activities by a sample of 19 RCH nurses

Activities	N	Mean Hour:min	Median Hour:min	95% confidence interval Hour:min	
				**Lower**	**Upper**

Case Management	8/19	00:29	00:19	00:04	00:54
Activities linked to vaccination (administration of EPI vaccines, IPTi, vitamin A, recording in Health Management Information System)	19/19	01:30	01:34	01:12	01:49
Maternal health (IPTp, Antenatal care, family planning)	18/19	00:59	00:45	00:31	01:26
Other (non contact productive-cleaning, day supplies preparation)	19/19	01:10	01:06	00:50	01:39
RCH nurse total time at facility	19/19	07:09	07:19	06:41	07:38
Overall non-productive time	18/19	03:06	01:06	02:14	03:58

Note: Time and Motion study of sub-sample of 24 facilities, 5 of which had no vaccination activities during researchers' visits

When the health workers were asked how could the services be improved, the suggestions given included increasing the number of employees, better maintenance of buildings, providing more working equipment and improving the availability of drugs (Table 5).

**Table 5:** Suggestions to improve services

Description	1^st ^response (N = 115)	2^nd ^response (N = 98)
	**%**	**%**
Increase number of employees	58 (n = 67)	18 (n = 18)
Maintenance of buildings	13 (n = 15)	2 (n = 2)
Provide more working equipment	11 (n = 13)	12 (n = 12)
Improve availability of drugs	6 (n = 7)	16 (n = 16)
More training for employees	8 (n = 9)	19 (n = 18)
Salary Increase	0 (n = 0)	27 (n = 26)
Other (for example staff housing, ambulance, improve laboratory)	4 (n = 4)	6 (n = 6)

### Workload perception

Half (8/15) of the nurses in the control areas were apprehensive that adding a new intervention was perceived to increase work load given small number of health workers at a facility. However they said they were ready to implement a simple new intervention due to expected benefits in saving lives; being administered jointly with already ongoing services and if it is a national policy. For example one nurse was quoted as saying:

"Although the intervention reduces malaria problem and children death, it will be difficult to implement if staff are not increased. I expect slight increase in work time as there is something additional because we are few staff". (In-depth Interview, Senior Public Health Nurse Grade B, 55 years old).

It is shown here that the health workers skepticism was not due to the nature of intervention but the health system bottlenecks.

In contrast, in places where an actual new malaria preventive intervention was jointly implemented with vaccination, nurses reported minor changes in work schedules in terms of requirements to document drug use and this was done within the usual working hours. In a facility where IPTi was being implemented, a nurse said:

"I am happy to execute IPTi as it is part of my responsibility. It has not come as a new work because the IPTi drugs are jointly administered with vaccine and does not need a separate planning. Since the drug prevents malaria, it has reduced children coming to seek care and has reduced workload. Before IPTi, all children from the area came on one day for vaccination, but the IPTi implementer advised us to do it by hamlet, this has worked out very well". (In-depth Interview, Maternal and Child Health Assistant nurse, 38 years old).

### Supervision

Although 84% of facilities had been visited by supervisors in the six months preceding the survey, only 13% (17/110) had received five or more visits and 49% had only received one or two visits (Table 6). Case management was observed in 20% of the visits, but the Health Management Information System forms had been completed to document the visit in 82% of the visits. Approximately 2/3 (62%, n = 69/111) of health staff found supervision visits helpful for reasons including bringing supplies, identifying expired drugs, following up on policy implementation, helping to identify problems and provide solutions, and provision of on the job training. There were also some negative experiences, including dissatisfaction with the supervision quality in 24% (26/105) of clinics because supervisors spent minimal amount of time at facilities, and infrequent visits. Some supervisors were thought to be incompetent or uninterested with the problems of the facilities. On occasions, supplies were not brought on time or drugs which had already expired were delivered. Some (15% (15/105)) of the health workers complained that the supervision was not supportive as it only engaged with the person in charge and did not provide direct feedback to other health workers. In about a third (32% (33/105)) of clinics, the respondents mentioned that some supervisors were unfriendly, made false accusations, lacked respect for clinic staff and failed to provide moral support. Nevertheless, the overall feeling was that supervisory visits were helpful.

**Table 6:** Supervision* activities in primary facilities

Number of times a facility is visited by a supervisor in last 6 month	Percent
0	16 (n = 21)
1-2	49 (n = 64)
3-4	22 (n = 29)
5+	13 (n = 17)

*Supervision visits: Average 2.2 and median 2

## Discussion

The documented low number of health workers assigned to rural health facilities and absenteeism in this study are comparable to other findings from Tanzania and elsewhere [11,30,31]. However, while other studies presented individual problems related to human resources productivity, capacity or incentives packages [32-39], the current study has brought them all together to show multifaceted nature of the human resources problem. These findings have serious implications for health service provision in southern Tanzania given that no more than one-fifth of the number recommended by the Ministry of Health's own guidelines were actually employed; of those employed, about half were absent from their duty station on the day of our survey; over half of the nursing staff followed during routine vaccination days were non-productive for at least three hours of the working day; and that supervision visits by district health staff to peripheral health facilities were infrequent and of variable quality.

The Ministry of Health established recommendations for staffing levels by interviewing key informants, observational studies and consultative meetings with staff in all levels of service provision [24]. The final criteria for staffing levels were based on the type of services provided, the type of health facility and the number of patients anticipated.

The norms might be appropriate for some places (e.g. urban dispensaries with a high utilization rate) but for others not (e.g. rural remote facilities covering a relatively small population). This may explain why the study identifies both time shortages and an inefficient use of available staff time. Accounting for service demand is crucial as utilization is likely to differ between remote facilities with lower population densities and few users compared to urban facilities with high population densities.

We found that only 14% of nurses' and 20% of clinical staff positions had been filled, lower than the national average of 35% [40]. We noted marked variation in staffing levels between the districts in our project area. The particularly marked lack of staff in rural settings has been documented previously [8] and results in service delivery being predominantly provided by untrained health workers. Mæstad suggested possible incentive schemes to attract trained people to work in rural areas [2]. "Pull incentive packages" could involve provision of hardship allowances, housing, improved management, local recruitment or clear career development plan; "push incentives" could involve implementation of coercive measures such as bonding, in which health workers are obliged to serve in rural areas for a number of years upon completion of internship. Testing how well such incentive schemes work in developing countries needs to be given priority.

Inadequate staffing levels were compounded by a high level of absenteeism which is not acceptable as it reduces access to services. Approaching a third of all employed staff were absent from their work place, resulting in only about 12% of the recommended staff actually being available at the health facility. Improved health services management is required to reduce the health workers in rural facilities being pulled in different ways - to attend seminars, to collect their salaries and sometimes vaccines or other supplies from the district capitals. Such distractions further undermine their ability to provide services. However, despite understaffing, the nurses in primary facilities did not appear to be overworked, suggesting that for preventive care there is a lack of balance between service supply and demand compared to recommendations of the Ministry of Health and Social Welfare and the internationally set requirement to attain the health Millennium Development Goals of 2.5/1000 health workers per population. Where nursing staff had been employed and were available on site in primary facilities, a surprising amount of time was non-productive, with over half the nurses being unproductive for at least three hours on a vaccination clinic day, considered to be the busiest time of the week. As observed and documented by researchers during our study, the variation in productivity was largely a function of patient flow compounded by lack of management: when patients were not present, nurses lacked the initiative to undertake other activities like filling HMIS forms or doing outreach clinics. The possible explanation could be the presence of untrained staff in primary facilities. This has an impact on quality of some services that require trained health workers for example maternal health and major issues related to HIV or non-communicable disease problems [41]. Patients in most instances value and search for services that they perceive to be of better quality. They could by-pass primary level facilities and seek care directly from higher level facilities perceived to have high quality, leading to loss in functionality of referral systems [42,43]. The consequence could be underutilization of lower level facilities, overload of hospitals as seen here and cases of high out-of-pocket payments for use of private facilities [44,45]. This is likely to be particularly detrimental for the poorest, increasing poverty through spending more than the limited resources available for basic needs. To increase access and client confidence for health service requires better availability of skilled health workers, improved service management, and support to reduce absenteeism.

In the decentralized Tanzanian health system, the district Council Health Management Team (CHMT) is responsible for the health services provided in its district. Those persons in-charge of primary facilities have a role in overseeing the day to day activities of their facilities and communicating with CHMTs on various requirements related to drugs, supplies and equipments. The CHMT members are supposed to visit each facility on a monthly basis to supply commodities, review HMIS data and support front-line staff. We found that such supervisory visits were infrequent and not always supportive. Adequate supervision could reduce absenteeism and mitigate some of the factors that reduce health workers' productivity [46]. However, CHMTs face genuine challenges in providing supportive supervision to peripheral health facilities. Many CHMTs plan a monthly supervision schedule, often found posted on their notice boards, but find it difficult to keep to it (personal observations and communications with District Medical Officers in rural districts). Competing interests lead CHMT members to attend training seminars, after which they are obliged to train front-line health workers, taking the latter away from their duty stations. Molyneux and others recommended more training in health facilities and fewer seminars in district head quarters in order to increase health workers' time for patient care and to increase the relevance of the training [47]. Another reason for failure to perform supervision and execute other duties on a timely basis is delay in disbursement of basket funds to the districts from the Ministry of Health and Social Welfare [*Personal communications with DMOs of Lindi Rural and Nachingwea in November, 2008*]. Additional local factors, such as the breakdown of vehicles and unavailability of fuel, compound the situation. In addition these same people are required to manage the HMIS, look after visiting officials and health stakeholders, who often arrive at very short notice, and to contribute directly to service provision in their districts. The distribution of paperwork such as guidelines and checklists is not enough to effect change: these needs to be complemented by agreed set of priorities, budget, follow-up, audit and feedback to lead to changes and influence performance [48]. Integrated supervision has been proposed to improve the efficiency of supervision visits as part of Tanzania Essential Health Intervention Programme (TEHIP), and this is worth taking forward [49]. Improved supervision is likely to require timely disbursement of funds, sufficient staff, prior notification of visits, appropriate training for supervision and improved supervision of CHMTs by regional and national level staff.

Our study may help those formulating polices to alleviate human resource problems. The number of health workers can be increased by promoting the WHO approach to recruit and train local people, residents of respective cultural zones within a country, and also to use mid level providers [50]. This will orientate health worker training and development of career incentives to encourage service in rural and disadvantaged areas to counteract the tendency of health workers to cluster around cities. The application of health worker management strategies through supportive supervision, improved supply of essential goods and integrated on the job training could reduce absenteeism and non productivity [46].

There were methodological limitations associated with this study. The facilities and health workers included in the time and motion study were purposively sampled. Nevertheless we believe they were representative of health facilities in the area. The time and motion study did not include private providers, where productivity patterns may differ from government providers. Although the time and motion approach is considered a gold standard in measuring health workers time use [51], it is subject to the so-called Hawthorne effect where what is being observed changes as a result of being observed. However this would likely result in positive bias [52], meaning that the documented productivity is higher in health workers under observation. We suspect the extent of this bias was reduced by the fact that interviewers carried out the time and motion study after they had spent several days at the facility, so that health workers had got used to their presence, and they used PDA technology which is less conspicuous than clipboards and pens. Another way in which the time and motion study may have over-estimated the productivity of health workers is that the study was done on the busiest day of the week, when vaccination activities were taking place.

## Conclusion

We have documented a shortage of front-line health workers, a high level of absenteeism and low productivity of existing health workers. Long-term investment in the Tanzanian health work force will be required to achieve adequate staffing levels. CHMTs require strengthening so that they are better able to conduct supportive supervision and there is a need to make health workers accountable to their supervisors and to the community. Improved management, service integration and staff incentives should enable health workers to perform better.

## Abbreviations

CHMT: Council Health Management Teams; HMIS: Health Management Information System; PDA: Personal Digital Assistant; MoHSW: Ministry of Health and Social Welfare; DPT: Diphtheria Pertussis Tetanus; EPI: Expanded Programme on Immunization; IPTi: Intermittent Preventive Treatment in infants; RCH: Reproductive and Child Health.

## Competing interests

The authors declare that they have no competing interests.

## Authors' contributions

FM conceived the idea and participated in the design of the study, coordinated data collection, conducted the analysis and writing the manuscript. JS helped develop the idea, study design, analysis, writing and interpretation. GH participated in the design of the study, provided technical support and contributed to the manuscript preparation. KW contributed to the manuscript preparation and interpretation. CM, KS contributed technical support and writing the manuscript. HM, MT provided technical support. DS participated in the design of the study, coordinated the study, data analysis and interpretation. All authors read, commented on and approved the manuscript.
